# Encounters between people with mental health and/or substance use problems and ambulance clinicians: a qualitative interview study in Norway

**DOI:** 10.1186/s12888-026-08546-4

**Published:** 2026-08-21

**Authors:** Lin Eik Ulvøy, Jorun Rugkåsa, Trine Møgster Jørgensen, Solveig H. H. Kjus, Kristin Häikiö

**Affiliations:** 1 Department of Nursing and Health Promotion, Oslo Metropolitan University (OsloMet), Oslo, Norway; 2 Health Services Research Centre, Akershus University Hospital (AHUS), Lørenskog, Norway; 3 Faculty of Health Sciences, VID Specialized University, Oslo, Norway

**Keywords:** Mental health problems, Substance use problems, Emergency medical services, Ambulance clinicians, Social stigma, Coercion, Personal autonomy, Patient-centered care, Communication, Norway

## Abstract

**Background:**

Interactions between ambulance clinicians and people with mental health and/or substance use problems are frequent, and international studies show that these encounters are often shaped by stigma, power dynamics and challenges to patient autonomy. However, there is limited research exploring patients’ experiences of ambulance services in the Norwegian context. This study addresses this knowledge gap.

**Methods:**

This qualitative study was based on in-depth, semi-structured interviews with 23 people with mental health and/or substance use problems recruited from primary and specialized healthcare services and service user organizations. The data were analyzed using reflexive thematic analysis, with input from a multidisciplinary stakeholder group throughout the research process.

**Results:**

Participants reported varied experiences in their encounters with ambulance clinicians. Prior interactions with other healthcare services strongly shaped their expectations of, and willingness to seek, emergency care. While some described professional and reassuring care, others experienced patronizing attitudes toward conditions perceived as self‑inflicted. Several participants also reported inadequate physical health assessments and treatment, and many felt excluded from decision‑making processes due to limited information, involvement, and respect. These experiences influenced their sense of safety, autonomy, and trust in emergency services.

**Conclusions:**

In Norway, people with mental health and/or substance use problems described several challenges in their encounters with ambulance services, including stigma, patronizing attitudes, inadequate physical healthcare, and limited patient involvement. These findings highlight the need for improved competence in mental health and substance use and clearer communication practices to promote more equitable and respectful ambulance care.

**Supplementary Information:**

The online version contains supplementary material available at https://doi.org/10.1186/s12888-026-08546-4.

## Background

Emergency medical services play a central role for people in acute mental distress. Calls to an emergency medical communication center (EMCC) may come from patients themselves, relatives, the public, healthcare personnel, or other emergency services [1]. Based on the call, ambulance clinicians (ACs) may be dispatched to assess the patient’s condition, triage the patient, and determine the need for further treatment. In these processes, often as part of intense or dramatic situations, interactions between patients and ACs can unfold in different ways.

Norwegian studies have shown a high prevalence of substance use problems among psychiatric patients, indicating that mental health and substance use problems often coexist and are difficult to separate in practice [2].

Several international studies have explored how people living with mental health and/or substance use problems/disorders (hereafter MH/SUD) experience their encounters with ambulance clinicians [3, 4]. Ferguson et al. interviewed men in Australia who had accessed ambulance services for MH/SUD [4]. The authors reported that the patients appreciated ACs who demonstrated professionalism, compassion, and effective communication, making them feel heard and respected. Results from another Australian interview study indicate that when patients receive empathy, reassurance, and clear advice from ACs, some feel sufficiently supported to choose not to be transported to hospital [5]. An interview study of young people in the UK reported that the quality of care from ACs was perceived as inconsistent and dependent on the individual clinician [6]. Together, these studies suggest that differences in patient experiences with ACs are often linked to perceived differences in these clinicians’ communication skills, empathy, and willingness to involve patients in decision-making processes [7, 8], as well as to broader systemic challenges, such as limited follow-up and care pathways for MH/SUD [9]. Further, when ACs are calm and acknowledge suffering, patients feel seen and taken care of, while a lack of compassion may lead to feelings of exclusion and powerlessness [9]. The perceived quality of care appears to be strongly linked to the individual clinician’s experience and attitude, while structural challenges such as inadequate training, weak collaboration with mental healthcare, and unclear rules for non-transport further complicate encounters [8, 10].

A growing number of qualitative studies have explored ACs’ experiences of their role in encounters with people with MH/SUD. These studies emphasize the increasing complexity and frequency of such cases in emergency medical services and show that these encounters are perceived by ACs as ethically demanding and emotionally taxing [7, 11–14]. ACs also emphasize that respectful and empathetic communication is essential to establish trust with individuals in a crisis. Studies have shown that ACs often feel unprepared to manage mental health crises, approach such situations primarily as acute physical emergencies, and experience uncertainty about their role [11–13]. The literature highlights the need for competence development in mental illness and communication skills, as well as improved inter-service collaboration and organizational support to reduce variability in care quality [11–13].

Ambulance call-outs to people with MH/SUD experiencing a mental health crisis typically occur in situations involving suicide risk, overdoses, or severe episodes of psychosis, where ACs must rapidly assess the patient’s condition and need for further treatment [15–17]. ACs called out to people with MH/SUD in mental health crises typically provide care involving situational control, patient stabilization and facilitation of further treatment [18]. It follows that these situations may affect patients’ self-determination and autonomy.

Patient autonomy is a fundamental principle in healthcare and refers to an individual’s right and ability to make informed decisions about their health and treatment [19]. To promote autonomy and self-determination, healthcare ethics emphasize informed consent and patient participation. In acute situations, this can be undermined by time pressure, which can lead to uncertainty and inaccurate assessments of risk and decision-making capacity. Relational perspectives on autonomy further emphasize that patients’ opportunities to exercise self-determination are shaped by communication, trust, recognition, and power relations in encounters with healthcare professionals [20, 21]. This may be particularly relevant in prehospital encounters involving people experiencing mental health crises and/or substance use-related problems, where questions of involvement, consent, decision-making capacity, and coercion often arise.

ACs operate under national regulations that define their scope of practice, including the provision of emergency care without consent in urgent situations [22]. They lack the authority to decide on direct admissions to inpatient care or use coercion under the Mental Health Care Act [22], The best way to manage the transportation of patients who resist care is subject to debate [23]. When patients resist care, the police hold the legal mandate to enforce compliance, and may be engaged to safeguard crew safety. However, their presence may make the encounter feel more coercive and amplify the patient’s experience of stigma [14]. Bergem et al. suggest that the absence of a clear national policy on coercion in mental health situations and an unclear regulatory framework lead to widespread use of coercion and underscore the need for clearer guidelines and recognition of ACs as ethical actors [14].

There is a lack of evidence to guide best practices in prehospital emergency care for people living with MH/SUD in the Norwegian context. Therefore, the aim of this study was to explore how people in this group experience encounters with ACs in acute situations in Norway. The study focuses on experiences related to mental health and/or substance use problems, which in prehospital emergency care frequently coexist and are often difficult to separate. By drawing on patients’ experiences in the Norwegian emergency context, this study complements clinician-focused research and highlights specific aspects of communication, involvement, and physical healthcare that may inform competence development and future guidelines.

## Methods

### Design

In view of the study aim, we adopted a phenomenological approach and used semi-structured qualitative interviews to gain insight into participants’ subjective experiences. Reflexive thematic analysis was used as the analytic approach due to its exploratory and flexible nature, allowing for an inductive examination of patterns of meaning across participants’ accounts [24]. This study adheres to the Consolidated Criteria for Reporting Qualitative Research (COREQ) [25].

### Setting

The Norwegian health service is organized into two levels: the municipal primary health service and the state specialist health service. ACs operate across levels, and ambulance missions are coordinated by an EMCC. This implies that people living with MH/SUD may encounter ACs in different contexts, such as at home, in public settings, while receiving municipal services, or in relation to admission to specialist emergency psychiatric units. In Norway, emergency medical services are publicly funded and organized through four regional health authorities, which comprise nineteen health trusts in all. The regional health authorities vary considerably in population, geography, and demographics, which may affect proximity to services.

Ambulances are staffed by healthcare professionals with national authorization, including emergency medical technicians, paramedics, and nurses, who operate independently under procedures defined by each health trust. Emergency calls are routed through the national emergency number 113 to one of sixteen EMCCs, where trained healthcare personnel assess and coordinate resources. The emergency services include ground, sea and air transport, and are integrated with both primary and specialist healthcare. All prehospital emergency services are provided free of charge to patients [26].

### Sample

This study was conducted in the South-Eastern and Western Norway Regional Health Authorities. This geographical variation was intended to include participants with experience from different health regions and to ensure diversity in participant backgrounds and service experiences.

Participants were recruited using a combination of purposive and snowball sampling to ensure variation in experiences and background characteristics. The first author (LEU), a female PhD student with 28 years of experience as an AC and prior experience in qualitative interviewing, contacted five municipal residential treatment facilities, five psychiatric units under the specialized healthcare services, and three service user organizations providing care for people living with MH/SUD. Staff members were asked to invite eligible service users to participate in the study. Initial contact with potential participants was facilitated by staff members or organizational leaders, who assisted in identifying individuals who met the inclusion criteria.

Inclusion criteria were being aged 18 years or older, having current or previous experience with mental health and/or substance use problems, and having had previous direct encounters with ACs. All participants had to be assessed as competent to provide informed consent to take part in research. This initial assessment of capacity to consent was conducted by staff members or organizational leaders who knew the participants and referred them to the study.

Individuals who were found eligible, agreed to participate, and consented to share their contact details were contacted by the researcher, who invited them for an interview at a time and location of their choosing. Before each interview, the researcher assessed whether the participant understood the purpose of the study, their rights, and what participation entailed, in line with research ethics guidelines. Recruitment and interviews continued until sufficient information power was reached. According to Malterud [27], information power implies that the more relevant information the sample holds, the lower the number of participants needed.

Twenty-five participants agreed to participate, although two did not complete the process. Information power was assessed after 10 and 20 interviews. During the later stages of data collection, participants’ accounts increasingly repeated similar experiences and perspectives related to communication, involvement, and perceptions of care in encounters with ACs. After 23 completed interviews, the data were therefore considered sufficiently rich and informative to address the aims of the study [28].

The final sample consisted of 13 male and 10 female participants, with an age range from 20 to 80 years. Participants were recruited from specialized healthcare services (*n* = 4), municipal healthcare services (*n* = 2), service user organizations (*n* = 12), and via snowball referrals (*n* = 5). Data on diagnoses was not collected, but participants talked about a variety of mental health and substance use problems, such as anxiety, depression, posttraumatic stress, dissociation, and psychosis, and use of alcohol, opioids and amphetamines.

### Development of the interview guide

An interview guide (Appendix 1) was developed based on a combination of existing literature, clinical experience and input from the multidisciplinary stakeholder group. The group included one person with lived experience, a family member of a person living with MH/SUD, two ACs, a psychiatrist, a physician, a lawyer, and a representative from the Norwegian Council for Mental Health. The guide was piloted in a single interview, which was deemed successful and was included in the final dataset without any revisions needed.

### Data collection

Data were collected through individual, semi-structured interviews conducted by LEU. The interviews took place between July and December 2023.

The interview guide was used flexibly, allowing the interviewer to adjust language, pace, and phrasing to accommodate what participants themselves emphasized as important. Interviews were tailored to the participants’ preferences, including location and the option for breaks. Participants were invited to suggest the location of the interview, which resulted in a wide variety of settings, including ambulance stations (*n* = 3), healthcare facilities (*n* = 14), service user organizations (*n* = 3), a hotel room (*n* = 1), outdoors (*n* = 1), and a private vehicle (*n* = 1). Conducting interviews across varied settings may have influenced the depth and openness of participants’ accounts. While allowing participants to choose the location probably increased comfort and a sense of control, some environments (such as ambulance stations, vehicles, or outdoor areas) may have introduced distractions or reduced privacy, potentially shaping what participants felt able to share. These contextual variations may also have influenced the depth, focus, and form of participants’ accounts across interviews, and this has been acknowledged as one of the study’s methodological limitations.

To ensure confidentiality, the researcher conducted the interviews in a manner that prevented others from overhearing the conversations. One participant requested the presence of a trusted support person, and two cohabiting participants chose to be interviewed together. The remaining 20 interviews were conducted individually. Each participant was interviewed once.

Participants were informed before the interviews about the researcher’s background as an AC and PhD student. No prior relationships existed between the interviewer and participants. Several participants expressed that the interviewer’s background gave them a sense of being understood and taken seriously, particularly when describing complex or stigmatizing experiences. All interviews were audio recorded using the “Nettskjema Dictaphone”, a secure application for collecting research data. The recordings were encrypted immediately and stored directly in the Services for Sensitive Data (TSD), administered by the University of Oslo.

Recordings were transcribed with Whisper software and reviewed and corrected by a project assistant independently of the analysis process. The transcripts were not returned to participants for comments or corrections. All data were handled within TSD. The duration of the interviews ranged from 13 to 60 min, with an average of 34 min. Data were coded manually using Microsoft Word and Excel.

### Analysis

The data were analyzed using reflexive thematic analysis, following Braun and Clarke’s six-phase approach [29, 30]. This method was chosen for its flexibility and suitability for exploring lived experiences and meaning-making processes. The analysis was iterative and reflexive, informed by repeated readings of the transcripts and ongoing reflexive notes.

In phase one (familiarization with the data), LEU and KH listened to the audio recordings and noted initial impressions, after which LEU read the transcripts multiple times to become closely familiar with the content. In phase two (generating initial codes), LEU generated initial inductive codes across the dataset, coding the material line by line to identify recurring ideas, experiences, and patterns.

In phase three (searching for themes), similar codes were compared and grouped across interviews, and potential themes were developed through iterative refinement. During this process, codes were merged, split, or discarded based on their analytic relevance. In phase four (reviewing themes), the developing thematic structure was discussed and refined through reflexive dialogue between LEU and KH, as well as within the whole author group. Input from the stakeholder group was consultative and used to support reflexive dialogue in the research team rather than to validate coding or determine themes. In phase five (defining and naming themes), the revised themes were discussed among all authors to ensure clarity, relevance, and coherence. In phase six (producing the report), the final themes were the final themes were integrated into a coherent analytical narrative, and the results were written up as an article. Examples of the analysis process are presented below in Table 1.

**Table 1:** Examples of the analysis process from data to themes

Transcript	Codes	Subthemes	Theme
Interviewer: Is there anything I haven’t asked about, something you want to say you think is important? How should the ambulance staff actually improve in these situations? Eva: I think it’s a lot about knowledge really, how much knowledge they have about various disorders and symptoms. I have complex PTSD as one of my diagnoses, and one of the things I struggle with the most is dissociation. While I have flashbacks, I dissociate, and then maybe people can’t make contact with me. I’ve found that when I’ve come back to the here and now, they look very confused and they’re like: “What was that?” “I’ve never seen that before.” They don’t know what to do about it, or it seems like they’ve hardly heard of what dissociation is, you know.	Inadequate training on PTSD/C-PTSD Lack of knowledge about dissociation Uncertainty when handling non-psychiatric presentations in prehospital care	Limited competence on mental health conditions Lack of preparedness for complex mental health conditions/ symptoms	Inadequate physical healthcare

Reflexive notes were taken by LEU throughout the interviews and analysis process to document personal reflections and potential biases. These notes were included in the discussions and interpretations during each phase of the analysis. In view of the interviewer’s extensive background as an ambulance clinician, we acknowledge that this positionality may have influenced data generation and interpretation. Participants may have adjusted their comments in ways that reflected social desirability or they may have hesitated to strongly criticize ambulance services. At the same time, the interviewer’s professional experience may have facilitated rapport during interviews and enabled deeper contextual understanding during analysis. These potential positive and negative influences were actively reflected upon throughout the study, and reflexive notes were used to critically examine how the researcher’s assumptions and prior knowledge could have shaped analytic decisions. The participants have been given pseudonyms to illustrate the variation in quotes.

## Results

Through the analysis, two main themes were identified:

- Previous healthcare experiences shaped expectations of future encounters with ambulance clinicians
- Experiences of ambulance services, with the following subthemes:
  - Feeling safe and being involved
  - Patronizing preconceived notions of “self-inflicted” conditions
  - Inadequate physical healthcare
  - Lack of self-determination, involvement and respect

### Previous healthcare experiences shaped expectations of future encounters with ambulance clinicians

Participants described how previous negative experiences of emergency wards, mental health services and other healthcare providers could reduce their trust and make them reluctant to seek help in later crises, including situations where ambulance care might have been needed. Eva (aged 20–30) described how responses she felt were uncaring or cold did not prevent her from self-harming, but instead made her more afraid to seek help:

Well, yes, I can understand that some of them choose to be consistently uncaring and cold-hearted, because they don’t want the patient to do those things just to get care. But for me, that doesn’t make me harm myself any less, but it could make me more afraid to seek help instead. (Eva)

Kristian (aged 50–60) similarly described how previous negative experiences affected his willingness to seek help, even for acute physical symptoms:

I was already reluctant to contact healthcare or call them. […] Even when I got appendicitis, at ten in the evening, I went to bed instead of calling an ambulance. (Kristian)

### Experiences of ambulance services

Many respondents expressed gratitude for the ambulance services. Most participants, however, mentioned both positive and negative experiences with ACs. While some described negative experiences such as feeling judged or excluded, several also emphasized positive encounters characterized by professionalism and reassurance.

#### Feeling safe and being involved

The sense of feeling safe and trusting ACs as professional healthcare workers was mentioned by some respondents. For example, Per (aged 30–40) explained:

I felt very reassured when they came to get me. When you see that red uniform, you think these are people who know their stuff. And then I automatically relax a bit. (Per)

A reason frequently given for feeling safe and being acknowledged by ACs was their sensitivity for, and ability to meet, the participants’ individual needs. Oddveig (aged 30–40) said that she appreciated the use of humor because it helped her get in a better mood. Stina (aged 20–30) described how ACs let her listen to music and choose the songs and volume in the ambulance, which gave her a sense of control and security. Per described calm interaction and clear information in the encounter, which facilitated his subsequent cooperation with healthcare providers.

Across the interviews, certain interactional strategies such as offering minor choices (e.g., choosing music), providing clear, anticipatory information, and using deescalating communication including light humor when appropriate, appeared to facilitate patient involvement and reduce stress.

#### Patronizing preconceived notions of “self-inflicted” conditions

Several participants said that the reason for the call-out provided by the EMCC clinician was crucial for how they were perceived by the ACs. Most felt that physical health conditions were considered more legitimate reasons for seeking healthcare than mental health and/or substance use problems. Many described feeling like a burden, saying that they felt that ACs viewed their substance use problem or mental healthcare needs as less legitimate or necessary than calls for physical conditions or injuries. Kristine (aged 50–60) reported having been collected by an ambulance several times for both physical and mental healthcare problems. She described that when mental health was her main reason for calling an ambulance, the ACs were often irritated and dismissive, expressed through their tone of voice, sighing, and non‑verbal cues, which she found emotionally overwhelming. She experienced encounters with ACs as indicating attitudes such as: “Oh no, not her again!” She said the ACs appeared discouraged and fed up, which she could tell from their tone of voice and the way they rolled their eyes, sighed, and avoided eye contact with her. This experience was also shared by Kjetil (aged 60–70). He said that situations involving “self-inflicted” harm, in contrast to typical physical health conditions, made him feel he was viewed as “stupid”, instead of ACs attempting to understand the underlying problem. He explained:

When it’s heart problems, it’s not something you do to yourself. Then I feel they respect me in a completely different way. If I come in after I’ve taken too many pills to end it all, I feel they look down on me. (Kjetil)

While Kjetil described this distinction through his own experience of ACs’ reactions to heart problems and overdoses, Kristian (aged 50–60) articulated the same divide more generally. Reflecting on how healthcare personnel might respond to a person with substance use problems, he described a distinction between what is understood as substance use-related and what is recognized as a “real illness”: “[…] either it’s drug-related, or it’s a real illness.”

A common perception among participants was that people with physical illnesses are treated with more respect by ACs than those with substance use and mental health problems. Even when their medical treatment was satisfactory, they viewed it as less meaningful if they were also not acknowledged and treated with dignity. However, in contrast to participants who felt dismissed, some emphasized how basic human kindness and professionalism fostered trust during the encounter. Siv (aged 20–30) expressed this in the following way:

[…] they make a difference just by being nice. (Siv)

#### Inadequate physical healthcare

Several participants described that their mental health and/or substance use problems influenced how ACs assessed and managed their physical health complaints. Truls (age 40–50) recounted how he initially received some pain relief, but that the staff gradually became more reluctant due to assumptions about his background:

[…] after a while they were kind of afraid to give me anything [pain medication] at all, because they thought I might be [too] affected by it, so I’d maybe get sick. (Truls)

#### Lack of autonomy, involvement and respect

Although negative experiences were prominent, several participants described encounters they felt were professional and supportive. However, for many participants, the experience of being excluded from decisions on their treatment was described as undermining their autonomy, self-determination and sense of being taken seriously. One participant emphasized that the lack of involvement and brief anticipatory information during transport reduced her sense of autonomy, increasing her stress and anxiety. Simple details about the destination and time could make a significant difference:

…it would be nice if they kind of said what they think might happen, like, if they think we need to go via the outpatient clinic, and inform me about that, or if we’re going straight to the hospital. (Stina)

Ole (aged 20–30) gave an example of a situation where he felt misled and disrespected, illustrating how a lack of involvement can erode trust and autonomy. Instead of informing him or involving him in a decision on voluntary or involuntary admission, the ACs misled him to cooperate on false premises:

[…] it’s an abuse of power, they’ve done it before […] By tricking me into the ambulance and saying I’m just going to see a doctor, and then they’ve admitted me without my consent. (Ole)

Åse (age 50–60) and Bjørn (age 50–60) both emphasized the importance of ACs being friendly, introducing themselves and asking how the patient was doing. Åse pointed out that people who have been treated badly before might be more alert, misinterpret ACs’ words, and lash out at them because they feel afraid and insecure. It would therefore seem that ACs need to be polite, respectful and considerate in order to strengthen patient autonomy and involvement.

Several participants described how their sense of autonomy and involvement could be further diminished when the police were present. Ole explained that the communication style of ACs changed to a more commanding and directive tone in these situations:

[…] yes, they get stricter […] It’s just: ‘You! Come here. Now. We’re leaving. (Ole)

Ole’s quote illustrates how contextual factors, such as the presence of law enforcement, can influence AC behavior and reinforce participants’ feelings of powerlessness and lack of involvement in decision-making.

## Discussion

This study explored how people living with MH/SUD experience their encounters with ACs. The participants described both positive and negative experiences and noted that prior encounters with healthcare services influenced their expectations for future services, thus affecting their willingness to accept the care offered by the ACs. Although participants reported diverse MH/SUD‑related experiences, the study was not designed to compare subgroups. Instead, we focused on shared experiences across acute situations, and the accounts showed substantial overlap in how participants described communication, stigma, and involvement in care. Although many participants emphasized feeling safe and involved, the main negative experiences were (1) patronizing preconceived notions, (2) inadequate physical healthcare, and (3) lack of Autonomy, involvement, and respect.

### Autonomy and self-determination

In encounters with ACs, some participants discussed positive experiences indicating professionalism and support, while others reported challenges that suggested a lack of respect for their autonomy and self-determination. Our findings suggest that participants’ opportunities to exercise autonomy were shaped through communication, information sharing, recognition and power dynamics in their encounters with ACs. Self-determination in healthcare means that all patients have the right to make informed decisions about their own care [31]. This requires sufficient information about the nature of the treatment, the potential risks and benefits, as well as the alternative treatments available [32]. Under the Norwegian Patient Rights Act (Lov om pasient- og brukerrettigheter), consent is a prerequisite for healthcare provision in most cases [33]. Although several respondents in our study expressed feeling safe and respected by the ACs as care providers, our results also indicate that people with MH/SUD may experience a lack of involvement in decision-making, patronizing attitudes, and limited information. This is well described in mental healthcare in general, but few papers have examined experiences with ACs [3, 34, 35]. These negative experiences can be understood as indicating a lack of respect for these patients’ self-determination and autonomy. The National Institute for Occupational Safety and Health defines autonomy as the right to self-determination and respect for the individual’s right to make informed decisions [36]. In this light, our findings suggest that limited involvement, insufficient information, and communication perceived as patronizing reduced participants’ ability to participate and make informed choices, thereby undermining their autonomy.

In mental health ethics, there is an underlying assumption that coercive measures are justified in certain situations when people with mental illness endanger themselves or others [37]. Although ACs have no mandate to use coercion in Norway, unlike the police and certain clinicians, previous studies have shown that they do quite often use force, but struggle emotionally when doing so and report feeling insecure about how to provide safe and efficient healthcare and transportation to people in a mental health crisis [14, 23, 38, 39]. Further, studies have shown that respectful communication and clear information are crucial for providing reassurance, reducing anxiety, and building trust in mental health crises [4, 40]. In order to strengthen patients’ autonomy and participation in healthcare decision-making processes, clear and transparent communication about the next step and active listening and brief explanations of aims and rights during emergency encounters are recommended [4, 40, 41]. In line with the findings in this study, previous studies have highlighted the importance of empathy, recognition, and clear communication in crisis management [4, 15, 41].

### Stigma and quality of care

Although stigma was not an explicit focus of the interview guide or a predefined analytic concept, participants’ accounts across the identified themes consistently reflected experiences of being judged, dismissed, or treated as less legitimate due to their MH/SUD. When the themes were considered in relation to one another, stigma emerged as a key issue for understanding participants’ experiences with ACs, particularly in relation to communication, involvement, and access to physical healthcare.

In our study, several participants reported being treated differently due to their MH/SUD problems. Some had experienced ACs arriving at the scene with preconceived attitudes and expectations. They expressed that the ACs’ actions, behavior, and decisions were unfair. Such behavior is described elsewhere as discriminatory [42]. According to Corrigan and Rao [43], people living with MH/SUD have long experienced discrimination and this phenomenon has been studied as stigma. The American Psychiatric Association defines stigma as “negative or discriminatory attitudes toward individuals with mental illness or substance use disorders among healthcare providers” [44].

Public stigma is well described in the mental health literature. Stigma towards MH/SUD is described in other emergency contexts, such as emergency wards, showing significantly lower ratings for respect and dignity, information, and involvement among patients who self‑report a mental health condition than those who do not [45, 46]. This is consistent with our findings of participants’ experiences of patronizing attitudes by ACs and the view of MH/SUD problems as different from “real problems”.

In addition to public stigma and stigma from healthcare personnel, self-stigma occurs when people internalize public attitudes and suffer negative consequences as a result [43]. Ferguson et al. reported that patients with mental health disorders already struggle with stigmatization in society and describe how these patients felt ashamed when calling an ambulance due to mental health problems, as they feared not being taken seriously [4]. This concurs with our results reporting that prior experiences influenced patients’ expectations and willingness to seek help.

Stigma has also been found to affect experiences of medical care, including care not directly related to MH/SUD conditions [45]. The effect of stigma on medical care was emphasized by the respondents in this study, particularly with respect to pain management and prioritization, where they experienced being undertreated and underprioritized due to their underlying MH/SUD diagnosis. Stigma and perceived discrimination can be reinforced through diagnostic overshadowing, where physical symptoms are mistakenly attributed to mental health or substance use disorders, and through premature closure, where clinicians end the diagnostic process too soon. Both patterns are often driven by distrust of patients’ descriptions of their symptoms [45], and both these processes may affect patients’ autonomy.

It is well documented that individuals with severe mental illness and substance use disorders have markedly reduced life expectancy, often 15 to 20 years shorter than that of the general population. A substantial proportion of these deaths is attributable to undiagnosed or insufficiently treated physical health conditions, making the failure to ensure adequate physical healthcare a critical challenge for quality of care [40, 45, 47, 48].

Participants’ experiences with ACs show that quality of care is not merely a matter of medical procedures but also the patient-clinician relationship. Both positive and negative experiences, therefore, call for specific strategies that should be integrated into training and practice: respectful communication in contrast to patronizing attitudes, high-quality and knowledge-based physical healthcare for people with MH/SUD, such as adequate pain relief, and respect for patient autonomy and self-determination by informing and involving patients even when they lack the capacity to consent. These elements are essential for strengthening trust, reducing stigma, and promoting patient-centered care [40, 49, 50]. Previous research shows that a lack of information and condescending communication can make patients feel ignored or deprioritized. This suggests a need for brief, standardized information about aims, next steps, and patient rights during the emergency encounter [40]. As seen in previous studies, our findings indicate that professional culture, operational pressure, and triage systems can amplify experiences of being overlooked or deprioritized [51, 52].

Our findings highlight the need for awareness of stigma and thus of the potential of unequal healthcare provision to this patient group in the ambulance service context. Stigma is known to impair quality of care and patient safety [49, 53, 54]. Prior research on paramedics and prehospital care reports a lack of knowledge among paramedics regarding mental health encounters and a feeling of being unprepared to help this patient group [6, 10, 13, 15, 55]. Our findings indicate a need for clearer guidelines and training for ACs in how to improve their communication skills and enhance patients’ autonomy and self-determination. Such competence building and supportive guidelines can lead to more equal treatment of physical conditions, regardless of MH/SUD problems, and thus improve the quality of ambulance care for people with MH/SUD problems.

### Implications for practice, education and system-level work

Based on our findings, interpreted in light of existing literature, it seems necessary to ensure that pain management protocols explicitly include patients with MH/SUD problems.

There is a need to strengthen education and training for ACs in how to communicate with people with mental health problems verbally, nonverbally, and by using de-escalation techniques [41]. Stigma and bias should be addressed through case-based learning and reflection [49, 56], as well as through simulation exercises with multidisciplinary teams, including the police. Our respondents noticed changes in AC behavior in the presence of law enforcement, which underlines the importance of strengthening collaboration and role clarification [41, 50]. Our results also indicate a need for clear routines for cooperation with law enforcement to maintain patient‑centered communication and reduce the risk of perceived coercion [7].

At the system level, better integration and adaptation of triage tools is advised, to enable them to capture the complexity of mental health symptoms, also in combination with physical health and substance use problems. Bergman et al. [17] state that current triage systems are often not used for mental health presentations, resulting in inadequate prioritization. Common guidelines should be developed for the police and ambulance services to clarify roles and responsibilities. To reduce the use of discretion and ensure more equitable services, attitudes among ACs should be followed up and linked to targeted measures [57].

### Strengths and limitations

The main strengths of this study include giving a voice to the perspective of a patient group that is often marginalized and underrepresented in research, and the involvement of a stakeholder group with diverse and relevant experience throughout the research process, as highlighted by Bee et al. [50]. The stakeholder group contributed to discussions on ethics, recruitment, development of the interview guide, and interpretation of findings, and one member with lived experience is a co-author, ensuring that various stakeholder perspectives are integrated throughout the presentation of results and discussion of implications. In addition, participants represented a broad variety of characteristics of people living with MH/SUD, encompassing diversity in service use, geographical location, gender, and age.

One limitation of this study is that recruitment was facilitated by healthcare staff or organizational leaders, which may have excluded individuals with more complex or critical experiences. This potential gatekeeping effect should be considered when interpreting the findings. This recruitment procedure may also have resulted in underrepresentation of individuals who were more acutely unwell, disengaged from services, or who had particularly negative or challenging experiences with healthcare services. Such selective participation may have influenced the breadth of perspectives included in the study and should therefore be considered when assessing transferability. Nevertheless, participants described a wide range of both positive and negative encounters, suggesting that the data still captured substantial variation relevant to the study aim. Another limitation is that we did not have access to patients’ diagnoses or medical histories, which makes it difficult to argue that they represent a variety of conditions. The absence of systematic information on diagnoses, symptom severity, or the timing of ambulance encounters increases the heterogeneity of the sample and limits our ability to assess potential differences between subgroups. This lack of diagnostic detail may affect the interpretability of nuances across MH/SUD presentations and should also be taken into account when assessing the transferability of the findings. This may indicate that the needs described are of a general nature, regardless of the specific diagnosis.

Another limitation is that interviews were conducted in a wide range of settings, some of which offered more privacy and stability than others. These contextual differences may have influenced the depth and openness of participants’ accounts and introduced some variability in the consistency of the data.

A further limitation is that two participants were interviewed together as a cohabiting couple. This dyadic format may have influenced how their accounts were constructed, including the possibility of co‑constructed narratives or reduced willingness to disclose sensitive experiences. This was considered during analysis and should be taken into account when interpreting the findings.

As stigma was not initially an explicit focus of the study, our interpretations rely on how participants chose to articulate their stigma experiences during the interviews. Nonetheless, the recurrence and consistency of stigma-related descriptions across participants indicates that this was an important aspect of their encounters with ACs.

## Conclusion

This study shows that people living with mental health and/or substance use problems experienced varied encounters with ambulance personnel. Several reported positive experiences, while others described poorer healthcare and discrimination due to stigma, reduced autonomy and compromised self-determination. Our findings identify the following challenges: previous negative experiences with healthcare services lead to expectations of similar experiences, condescending attitudes towards “self-inflicted” conditions, inadequate physical health treatment, and limited involvement in decision-making. On the other hand, several participants described positive encounters characterized by professionalism and supportive communication that provided reassurance. Future studies should further examine the experiences and perspectives of people with MH/SUD in relation to prehospital services and explore opportunities to enhance ambulance clinicians’ competence and develop clearer guidelines for their care for this patient group.

## Supplementary Information

Below is the link to the electronic supplementary material.

Supplementary Material 1

## Data Availability

The datasets generated and analyzed during the current study are not publicly available due to privacy and ethical restrictions.

## Declarations

### Use of artificial intelligence tools

Artificial intelligence tools (Microsoft Copilot, enterprise version) were used to assist with translation of participant quotes from Norwegian to English and language editing of the entire manuscript. All outputs were reviewed and verified by the authors for accuracy and appropriateness. The AI tool was used in a secure enterprise environment that does not share or store data externally, ensuring compliance with data protection and confidentiality requirements. The entire text has since been edited by a well-qualified native speaker of English.
