# Supplementary material for: Encounters between people with mental health and/or substance use problems and ambulance clinicians: a qualitative interview study in Norway
Source: BMC Psychiatry. 2026 Aug 21;26:722. doi: 10.1186/s12888-026-08546-4 (PMC13579872; doi:10.1186/s12888-026-08546-4)
Supplement: Supplementary file 1 [file 12888_2026_8546_MOESM1_ESM.docx]

**Appendix 1**

**Interview guide for mentally ill patients and/or patients with substance abuse disorders who need an ambulance.**

Welcome and introduction to the interview. Information about confidentiality, anonymisation and the possibility of withdrawing from the study. Do you have any questions before we begin?

1) Can you give an example of an incident where you needed an ambulance?
2) Were you consulted on questions related to your treatment?
   a. Level of treatment?
   b. Hospitalization or not?
   c. Who was involved in the process?
   d. Was coercion used?
3) Do you have any thoughts about the information that was given before, during and after the meeting with the ambulance?
4) Was there any information you missed in the actual meeting with the ambulance personnel?
5) Can you tell me about the process of meeting with the ambulance service? What is important to know for you in this type of meeting?
6) Can you say something about your experience of security/insecurity in the encounter with the ambulance personnel?
   a. Did you have confidence in those who came to you?
7) How did you feel that the ambulance personnel took into account what you communicated to them?
8) Do you have previous experience with the need for an ambulance?
   a. If so, how would you compare these meetings?
9) Do you think the experience would be different if you needed an ambulance for a somatic problem such as chest pain?
   a. In what way has the experience been different, or what do you think would have been different?
10) Do you think the meeting place with an ambulance has anything to do with the meeting with the ambulance personnel?
    a. home or public place.
11) Have you ever been picked up by both the police and/or ambulance?
    a. How do you experience being picked up by the police versus just an ambulance?

Concluding/Summing Up

12) What have you experienced in the encounter with an ambulance that you would like to highlight as particularly good?
13) What have you experienced in the meeting with the ambulance service that is not particularly good?
14) Is there anything I haven't asked about that you want to say something about?
